# Factors Associated with Work-Related Burnout among Corporate Employees Amidst COVID-19 Pandemic

**DOI:** 10.3390/ijerph19031295

**Published:** 2022-01-24

**Authors:** Lawrence T Lam, Mary K Lam, Prasuna Reddy, Prudence Wong

**Affiliations:** 1 Tung Wah College, Hong Kong, China; 2 Faculty of Health, University of Technology Sydney, Ultimo, NSW 2007, Australia; 3 Department of Health and Biomedical Sciences, RMIT University, Melbourne, VIC 3083, Australia; mary.lam@rmit.edu.au; 4 Faculty of Science, Engineering and Technology, Swinburne University, Hawthorn, VIC 3122, Australia; preddy@swin.edu.au; 5 Mental Health Association Hong Kong, Hong Kong, China; prudenceykwong@gmail.com

**Keywords:** work-related burnout, protective factors, risk factors, work environment, COVID-19 pandemic

## Abstract

Work-related burnout is common and has detrimental effects on employees in many industries. The current study aims to examine both environmental and personal factors that are associated with work-related burnout in a population of corporate employees who managed to retain their jobs amidst the global COVID-19 pandemic crisis. This cross-sectional survey served as the baseline data collection of a phase III wait-listed cluster randomised controlled trial (CRCT) of an intervention program on mental well-being at the workplace. Participants were recruited from six large-size corporations. Work-related burnout was assessed by the Maslach Burnout Inventory (MBI), and the Moos Work Environment Scale (WES) was used for evaluating the workers’ perspective on the workplace. Information was also collected on demographics and health behaviours. Burnout in this sample was prevalent with 60% of participants rated at a moderate to a high level on emotional exhaustion. Results from the multiple linear regression analyses suggested that different factors were related to different components of burnout. For example, age, work involvement, co-worker cohesion, and work pressure were associated with emotional exhaustion and depersonalisation while others were related to professional accomplishment. The overall results suggested that the work environment is of influential importance to the burnout of employees. However, although the study was conducted during the peak of the COVID-19 pandemic, the factors identified as relating to workplace burnout do not differ much from those identified in a crisis time. Implications of the results were discussed.

## 1. Introduction

Work is an essential part of our daily life. It has been estimated that on average, full-time workers in OECD countries spend about 37% of their time working in a normal day [1]. The positive and negative aspects, or harm and benefits, of work have long been recognized [2]. On the positive side, many benefits can be gained from work including, monetary rewards, personal development, sense of worth, self-esteem, status and prestige, and their effects on both physical and mental health [2]. On the other hand, some work-related characteristics have been identified as being associated with common health problems, particularly mental health [3,4]. In a large-scale study on the mental and physical health of a working population, Rose et al. found that work-related stress and fatigue were associated with mental health problems such as depression [5]. Review studies have also found that several work-related factors may be involved in the aetiology of clinical depression. These factors include work-related stress and burnout, job insecurity, the imbalance between effort and reward, workplace bullying, and a low sense of control over work [6,7,8,9]. Work-related burnout has also been identified as a contributing factor to absence from work due to long-term illness and thus the loss of productivity [10].

Burnout is classified as an occupational phenomenon, not as a medical condition, in the 11th version of the International Classification of Diseases (ICD-11). It is defined as: ”a syndrome conceptualized as resulting from chronic workplace stress that has not been successfully managed [11]”. Maslach and Jackson first attempted to evaluate burnout with the design of the Maslach Burnout Inventory (MBI) and observed three main aspects of burnout, namely emotional exhaustion, depersonalisation, and the diminishment of professional accomplishment and thus the formation of the MBI [12]. Since then, the MBI has been widely used for the study of burnout globally.

After years of research on the topic, Maslach concluded that burnout is an occupational-specific phenomenon and recognised that most of the research has been focusing on the human services area, particularly in the health care sector [13]. Understandably, due to the work nature and the demand for personal resources, a large number of front-line health care professionals are more vulnerable to burnout. However, burnout could also be easily found among workers in other industries such as childcare, education, social welfare, and media and communication [14,15,16,17].

In terms of the risk factors of work-related burnout, particularly at a time when the world was not drastically affected by a global crisis, such as the COVID-19 pandemic, many have been suggested and studied [18]. As summarised by Aydemir and Icelli, the risk factors for work-related burnout can be broadly classified into environmental and personal factors. The environmental factors include, but are not limited to, the physical environment, work overload, workplace unfairness and bullying, lack of control over work, and insufficient compensation or reward. For personal factors, some demographics and individual personalities may also contribute to burnout among workers [18]. Genetics was also proposed to play a role in the burnout syndrome of workers. Evidence on the association between gene variants and burnout is not strong, but is worth further exploration [19,20]. More recent studies have seen a gradual shift from the risk factors to protective factors in the workplace [21,22,23]. For example, in the work environment Anyfantis et al. found that interpersonal relationships and having a degree of freedom in work were protective against burnout among labour inspectors [23]. Hämmig also acknowledged that support from the direct supervisor in case of a problem at work and home could be a protective factor [21]. Personal factors, such as psychological resources, self-esteem, and the meaningfulness of work were also negatively associated with burnout at work [22,23].

In the past two years, due to the global crisis of the COVID-19 pandemic, the work life of the working population in many countries has been changed drastically. For some, the pandemic has caused the cessation of work because of the closure of businesses and redundancy, or as a result of restructuring and downsizing. Some workers may experience a change in their status, position, or a total departure from the industry in which they have been working for many years. The changes in the macro- and micro-work environments may pose new challenges to workers and affect modes of operation in many different industries. As a result, different influencing factors of work-related burnout may arise under this unusual circumstance. As part of a government-funded intervention program for enhancing mental health and wellbeing in the workplace, data on personal and environmental variables were collected at the baseline, post-intervention, and 3-month follow-up [24]. The current study aims to report both environmental and personal factors that are associated with work-related burnout in a population of corporate employees who managed to retain their jobs amidst the global crisis.

## 2. Methods

This cross-sectional survey served as the baseline data collection of phase III wait-listed cluster randomised controlled trial (CRCT) of an intervention program on mental well-being at the workplace. The full trial protocol had been published previously in 2019 by the author [24]. The baseline survey was conducted in May 2021 during the period when the global situation of the COVID-19 pandemic was still unstable, and Hong Kong was experiencing the 4th wave of attack. Institution ethics approval for the study was granted by the Human Research Ethics Committee of the Tung Wah College.

The sample consisted of adult employees of six corporations of a reasonably large size in Hong Kong with none of the participants facing employment uncertainty at the time of recruitment. These participants were recruited from these corporations through an announcement from the corresponding Human Resources departments. Participation in the project was voluntary without any coercion from the employers or the research team. On the other hand, the senior management of these corporations was strongly supportive of the project with the provision of time relief for their participating employees. Details on the recruitment process and the randomisation were reported in a previous article [24]. The health survey was conducted via an online platform purposely built for the project as part of the psychoeducation component of the intervention program. Participants, both in the intervention and control arms, were invited to complete the survey immediately before the randomisation process [24].

Work-related burnout was assessed by the Maslach Burnout Inventory (MBI) which, as aforementioned, was specifically designed to measure the extent of burnout in adults in the workplace [12]. The instrument consists of three main domains capturing three different aspects of the burnout phenomena in individuals. As suggested by the title of the subscale, emotional exhaustion measures the feeling that one is exhausted emotionally by work. Depersonalisation captures the state that the worker is impersonally responding to his/her clients or recipients of one’s service or care. Professional accomplishment measures the level of competence and achievement of the worker in providing services. This instrument was fully validated and has been widely used around the world for many years [25,26]. It has also been translated into many languages. Recent studies on the psychometric properties of the MBI suggested that the three-factor model fitted well with the data when compared to alternative models, with high internal consistency for all three subscales yielding Cronbach’s alpha values of 0.84, 0.87, and 0.88, respectively [25]. In this study, to provide higher power for the study, subscale scores were used for analyses.

For the environmental factors, the Moos Work Environment Scale (WES) was used for evaluating the workers’ perspective on the workplace [27]. The WES was designed for measuring the social environment of the work settings with three main underlying dimensions, namely: workplace relationship (involvement, cohesion, and supervisor support); personal growth (achievement, values, and professional growth); and management (communication, controls, and changes). In each subscale, a set of direct statements were presented to the respondent seeking a response by an indication of True or False as an endorsement. The endorsement of a statement in a direction demonstrating a positive view on the construct would gain a score of 1, otherwise 0 on the item. The raw scores of the subscales were calculated by the summation of all positive items and the raw scores were then converted to a standardised score ranging from 0 to 100. The WES was fully validated and standardised with normative data provided in the manual [27]. This study focused on five subscales for ease of interpretation and to accommodate for local workplace characteristics. These subscales included: involvement, co-worker cohesion, supervisor support, work pressure, and managerial control.

In terms of personal factors, data were collected on a range of variables including demographics, duration of employment in the industry, and whether the respondent had thought about resignation. Other information on health conditions and behaviours was also collated, including drinking, smoking, physical activity, and sick leaves.

Data were analysed using the Stata V17.0 statistical software program. Since participants were recruited from six corporations with different natures of business, to adjust for the clustering effect the dataset was pre-set with the *svy* command with a simple weight factor calculated as the inverse of the frequencies of each site. Descriptive statistics on the variables were presented with frequencies and percentages or means and standard errors. Bivariate analyses were conducted to examine unadjusted relationships between burnout and all variables of interest. The unadjusted associations between burnout and these variables were investigated using simple linear regression modelling analyses. For the inclusion of potential risk or protective factors in multivariate analyses, variables attaining a *p* ≤ 0.20 in the bivariate analyses were selected to be included in further analyses. Multinomial logistic regression analyses, using the lowest level of burnout as the referent group, were employed to examine the adjusted associations of the included variables. A backward elimination model reduction process, based on the adopted type I error rate as the decision criterion, was applied for achieving the most parsimonious model. As an exploratory study aiming to identify potential factors associated with the outcome, the interaction terms of significant variables retained in the final model were not considered. A type I error rate of 5% was adopted for testing the significance of each variable in the model.

## 3. Results

A total of 456 participants were recruited to the trial from six corporations of different natures of business including real estate, insurance, property management, and other industries. Baseline data collected prior to the randomisation of participants in the intervention and control groups of the CRCT were complete without any refusals to respond to the survey. The descriptive statistics on the demographic, health, and work-related variables, and the outcome variables, namely work-related burnout were summarised in Table 1. As shown, the average age of participants was 38.3 years (s.e. = 2.4) with slightly more females than males (54.6% vs. 45.4%), with the majority attaining an education level of a university degree or above (n = 370), and less than half were married (48%). For work-related variables, all participants worked full-time except three, with an average duration in the industry of 8.3 years (s.e. = 1.1) and about one-third (n = 143) could work flexible hours. The majority of participants were non-smokers (95.6%), light or non-drinkers (92.2%), and more than two-thirds exercised regularly (n = 342), with only a small proportion (8.2%) requiring three or more days of sick leaves in the past three months. In terms of the work environment measures by the Woo’s Work Environmental Scale, the mean standardised scores of nearly all domains were close to 50 with 48.2 (s.e. = 2.8) for work involvement, 51.3 (s.e. = 1.6) for co-worker cohesion, 54.3 (s.e. = 4.7) and 50.1 (s.e. = 2.1) for supervisor support and work pressure, respectively. Workplace management control was the only domain that scored the highest with an average standardised score of 60.5 (s.e. = 5.0). However, slightly more than half (52.7%) of participants indicated that they had contemplated resigning from their company in the past three months. Regarding the outcome measures of this study, namely work-related burnout, the average scores of the three domains including emotional exhaustion, depersonalisation, and professional accomplishment were 21.6 (s.e. = 0.4), 6.9 (s.e. = 0.4), and 27.8 (s.e. = 1.1), respectively. These represented about 60% of participants rated at a moderate (29.2%) to a high level (31.5%) on emotional exhaustion, about 45% on depersonalisation (moderate/high: 29.9%/15.4%), and only 32% on professional accomplishment (moderate/high: 20.9%/11.0%) following the classification provided by the scale authors.

The bivariate relationships between the three domains of work-related burnout and participants’ demographics, health and work-related variables, and the work environment variables were investigated with the results summarised in Table 2. As shown, with only adjustment for the clustering effect, a different set of variables were associated with each of the three domains. For example, age, education level, and working duration were related to emotional exhaustion but not the other two domains. On the other hand, intention to resign and supervisor’s support was associated with all three. (Table 2) These variables, with others that satisfied the pre-set selection criteria, were included in further analyses.

Results of the final models of the Multinomial logistic regression analyses on factors associated with the three domains of work-related burnout were summarised in Table 3. As shown, a slightly different set of variables was associated with different levels of the three burnout domains. For emotional exhaustion, three variables were retained in the final model, but were only significantly related to the high level of this domain of burnout. This included age, co-worker cohesion, and work pressure, with the former two negatively, and work pressure positively, associated with emotional xxhaustion (OR = 1.09, 95%C.I. = 1.05–1.13). These results suggested that an increase in age and co-worker cohesion was related to a decrease in the emotional exhaustion of workers, and an increase in work pressure was associated with an increase in emotional exhaustion. For depersonalisation, four variables were identified as being related to different levels of this domain, At the moderate level of depersonalisation, age, co-worker cohesion, and work pressure were significant in the same direction as with emotional exhaustion, having odds ratios of 0.95 (95%C.I. = 0.92–0.99), 0.98 (95%C.I. = 0.97–0.99), and 1.03 (95%C.I. = 1.00–1.06), respectively. At the high level of depersonalisation, a slightly different set of variables was observed, with age and co-worker cohesion remaining, and work involvement replacing work pressure. All three variables were negatively associated with the high level of depersonalisation with odds ratios of 0.88 (95%C.I. = 0.82–0.95), 0.97 (95%C.I. = 0.95–0.98), and 0.98 (95%C.I. = 0.97–0.99), respectively. In terms of the professional sccomplishment domain, two variables were retained in the final model, namely drinking and resignation intention. These variables were both positively related to the moderate level of the domain only with odds ratios of 2.87 (95%C.I. = 1.17–7.36) and 1.66 (95%C.I. = 1.10–2.51), respectively.

## 4. Discussion

This study aims to examine factors associated with work-related burnout among employees of corporations during the peak of the COVID-19 pandemic utilising the baseline data collected for a phase III wait-listed cluster randomised controlled trial of an intervention program on mental well-being at the workplace. In terms of the extent of burnout reported in this sample, the prevalence of 60% and 45% for a moderate to a high level of emotional exhaustion and depersonalisation, and only 32% in professional accomplishment, was higher than that reported in other working populations [16,17]. While there were some commonalities of potential factors such as age, the results suggested that different variables were correlated with different aspects of the burnout constructs. As expected, most of these significant variables are related to the work environment except those associated with the professional accomplishment domain of the burnout measure. For emotional exhaustion and depersonalisation, work involvement and co-worker cohesion were negatively related to these burnout measures, whereas work pressure was positively associated with both. These results were in line with the expected direction and consistent with the literature [21,23]. On the other hand, the gender difference in burnout has long been recognised and studied [28]. Results of the mate-analytical study revealed that females have a slightly greater propensity for emotional exhaustion than males, whereas males are somewhat more depersonalised than females [28]. However, the differences in terms of the effect sizes are not great. The results obtained from the current study showed no statistically significant differences on all three domains of burnout between genders, which could possibly to related to cultural differences, and is worth further exploration. The overall results suggested that the work environment is of influential importance to the burnout of employees. Another point worth noting is that, although the study was conducted during the peak of the COVID-19 pandemic, the factors identified as relating to workplace burnout do not differ much from those identified in a non-crisis period. As we all have experienced and are experiencing, the COVID-19 pandemic causes a lot of disruptions to daily life, and particularly to our work life. Employees of nearly all industries are inconvenienced by the restrictions to the workplace resulting in many working from home if their work nature could be adapted to a different environment. Others, particularly manual labourers, are facing a lot more hardship with work stops or even redundancy. For those who could retain their employment, such as those recruited in this study, while in general people were concerned about the overall health problem, when it came to work-related matters, the work environment played a predominant role in affecting workers’ mental wellbeing. Results may also be a reflection of employees’ views on work-related issues that have been ongoing for some time before the onset of the pandemic, given that the participants had been working for their companies for some time already.

In this study, a selected set of work environment domains were used based on the contextual requirements and suitability of these domains to the local work environment. Results of the study indicate that the work-related environmental factors associated with burnout are mainly concerned with work relationships, with the exception of work pressure. Work involvement reflects the extent that employees are devoted to their work, motivation, and excitement about working for the company. Co-worker cohesion, as suggested by the name of the domain, is concerned with harmonious and supportive mutual relationships among employees. It has long been shown that a positive work experience is conducive to stress reduction and promotes better mental well-being in the workplace [29]. As in all kinds of relationships, it has been well proven that a positive and constructive work relationship can have many benefits, including an increase in work efficiency, the enhancement of creativity, increased productivity, and the retention of quality staff [30]. Good retention of staff means the reservation of human resources and retainment of organisational and corporate knowledge and wisdom [31]. A work environment that values good relationships among colleagues and between supervisors and supervisees with opportunities for involvement for all in the workplace could help to create a stronger sense of belonging and loyalty. This could offer a buffering effect against the demands and stresses of work and may possibly reduce work pressure and other negative impacts. Another indication of work-related burnout is a reduction of the sense of professional accomplishment, reflecting that employees could not achieve what they otherwise would have been able to. Supervisor support and mentorship, as shown in the results of this study, is a significant protective factor in combating work-related burnout.

Some direct implications on organisational management and the promotion of mental wellness among workers may be drawn from the results obtained from this study, although no causal relationship can be implicated in this cross-sectional study. A few lessons can be learned that might be appliable to normal circumstances and more so in a crisis, such as the pandemic we are experiencing now. First, a good relationship between employers and employees, as well as among colleagues, could be a key to maintaining a healthy and positive work environment. Such a work environment provides many benefits to all parties as noted above. These benefits include protecting workers from burnout and promoting better mental well-being in employees. There are many possible strategies that employers would use to achieve a healthy and positive workplace. However, this is outside the scope of the current discussion. Second, work-related stress could likely be a result of relational issues and problems. A lack of involvement of employees and cohesion among colleagues is related to burnout, as shown in this study. The work-related stress and actual work pressure could also be associated with burnout, and could be a causal factor in workers’ intentions to resign, as suggested in the results obtained. Addressing issues in, and problems of, workplace relationships, and maintaining a healthy relationship between employers and employees with mutual respect should be considered a very important priority in all workplaces, particularly in the current situation.

As the baseline data collection of a CRCT, there are strengths and weaknesses identified. This is not a population-based study with recruitment using a random sampling technique for sample generation. Participants were recruited via the Human Resources departments of six large-sized multinational corporations voluntarily. These corporations covered a wide range of different industries and work natures, ranging from manual labourers to high-level executives. Hence, the sample captured many sectors of the local working population. This study utilised standardised and validated assessment instruments for all main variables of interest, thus minimizing measurement and interpretation biases. On the other hand, being part of the CRCT for an intervention program, there is a limited number of variables to be included as potential risk and protective factors. Hence, the factors identified to be associated with burnout might not be exhaustive. It is also worth noting that some variables are significantly related to the outcome measures in the bivariate analyses only marginally, with a *p*-value near 0.05. These variables have been included in the regression analyses. Given the sample size of the RCT and the selective number of potential risk and protective factors, the interpretations of the significance of these variables in the analyses are to be treated cautiously in terms of their theoretical and practical implications. Furthermore, as indicated by the health-related variables of the sample, it is a rather healthy and homogeneous group, thus the overall results obtained from the study might not be generalised to other populations with different health profiles. Finally, this study is an exploratory study intended to identify the potential risk and protective factors for work-related burnout. Future studies could be conducted to include other variables, such as work-related stress, and to further examine the potential interactive effect of these factors on burnout.

## Figures and Tables

**Table 1:** Descriptive statistics on participants’ demographic and other variables (N = 456).

Participants’ Characteristics	Frequency (%) or Mean (s.e.) ^a^
Demographics	
Age (years)	38.3 (2.4)
Sex	
Male	224 (45.4%)
Female	232 (54.6%)
Education level	
University or above	370 (64.5%)
Secondary and post-secondary	86 (35.5%)
Marital status	
Married	177 (48.0%)
Others	279 (52.0%)
Working duration (years)	8.3 (1.1)
Full time	
Yes	453 (97.4%)
No	3 (2.6%)
Flexible hours	
Yes	143 (36.3%)
No	313 (63.7%)
Health-related variables	
Regular exercise	
Yes	342 (80.6%)
No	106 (19.4%)
Smoker	
Yes	16 (4.4%)
No	436 (95.6%)
Drinker	
Moderate/heavy drinker	32 (7.8%)
Light drinker	411 (92.2%)
Sick days in the past 3 months	
Less than 3 days	419 (91.8%)
3 days or more	29 (8.2%)
Work environment-related variables	
Intended to resign	
Yes	244 (52.7%)
No	212 (47.3%)
Standardised score of work involvement	48.2 (2.8)
Standardised score of co-worker cohesion	51.3 (1.6)
Standardised score of supervisor support	54.3 (4.7)
Standardised score of work pressure	50.1 (2.1)
Standardised score of management control	60.5 (5.0)
Outcome variables	
Burnout—Emotional exhaustion	21.6 (0.4)
Burnout—Depersonalisation	6.9 (0.4)
Burnout—Professional accomplishment	27.8 (1.1)

^a^ Adjusted for the clustering effect.

**Table 2:** Correlation or mean difference (s.e.) and unadjusted associations between demographic, health-related, work environment-related variables, and burnout.

Participants’ Characteristics	Results on the Association ^a^		
Demographics	Emotional Exhaustion	Depersonalisation	Professional Accomplishment
Age	r = −0.25 *	r = −0.21	r = 0.18
Sex	µ_diff_ = −0.91 (1.30)	µ_diff_ = 0.44 (0.96)	µ_diff_ = 1.31 (1.47)
Education level	µ_diff_ = 3.58 (1.03) *	µ_diff_ = −0.91 (1.30)	µ_diff_ = 0.56 (0.58)
Marital status	µ_diff_ = 1.80 (1.18)	µ_diff_ = 0.02 (1.18)	µ_diff_ = −0.44 (0.81)
Working duration	µ_diff_ = −0.18 (0.05) *	µ_diff_ = −0.08 (0.06)	µ_diff_ = 0.22 (0.11)
Full time	µ_diff_ = 4.99 (0.43) **	µ_diff_ = 3.11 (0.46) **	µ_diff_ = 6.01 (1.16) **
Flexible hours	µ_diff_ = 1.75 (1.00)	µ_diff_ = −0.31 (0.59)	µ_diff_ = 1.86 (1.92)
Health-related variables			
Regular exercise	µ_diff_ = 4.65 (1.30) *	µ_diff_ = 0.93 (1.64)	µ_diff_ = −2.62 (0.61) *
Smoker	µ_diff_ = 7.27 (2.97)	µ_diff_ = 7.69 (2.92)	µ_diff_ = −0.69 (0.95)
Drinker	µ_diff_ = 2.27 (3.00)	µ_diff_ = 0.66 (1.51)	µ_diff_ = 3.65 (1.04) *
Sick days in the past 3 months	µ_diff_ = 9.24 (6.20)	µ_diff_ = 3.63 (1.54)	µ_diff_ = −6.01 (1.97) *
Work environment-related variables			
Intended to resign	µ_diff_ = −5.65 (1.53) *	µ_diff_ = −3.08 (0.88) *	µ_diff_ = 3.82 (0.34) **
Work involvement	r = −0.22	r = −0.25 *	r = 0.16
Co-worker cohesion	r = −0.26 *	r = 0.25 *	r = 0.08
supervisor support	r = − 0.18 *	r = −0.23 *	r = 0.16 **
Work pressure	r = 0.48 **	r = 0.37 *	r = −0.05
Management control	r = 0.13	r = 0.11	r = −0.13 *

^a^ Adjusted for the clustering effect. * *p* < 0.05; ** *p* < 0.01.

**Table 3:** Results obtained from the final model of the multinomial logistic regression analyses.

Variables Retained in the Final Model	OR (95%C.I.) ^a^	Significance
Outcome: Emotional exhaustion		
Moderate		
Age	0.96 (0.93–1.0)	t = −2.34, *p* = 0.079
Co-worker cohesion	0.99 (0.96–1.02)	t = −1.25, *p* = 0.278
Work pressure	1.01 (0.98–1.04)	t = 1.17, *p* = 0.306
High		
Age	0.92 (0.88–0.96)	t = −5.68, *p* = 0.005
Co-worker cohesion	0.98 (0.97–0.99)	t = −5.78, *p* = 0.004
Work pressure	1.09 (1.05–1.13)	t = 5.83, *p* = 0.004
Outcome: Depersonalisation		
Moderate		
Age	0.95 (0.92–0.99)	t = −3.49, *p* = 0.025
Work involvement	0.99 (0.98–1.01)	t = −0.40, *p* = 0.712
Co-worker cohesion	0.98 (0.97–0.99)	t = −7.99, *p* = 0.001
Work pressure	1.03 (1.00–1.06)	t = 3.05, *p* = 0.038
High		
Age	0.88 (0.82–0.95)	t = −4.65, *p* = 0.010
Work involvement	0.97 (0.95–0.98)	t = −6.40, *p* = 0.003
Co-worker cohesion	0.98 (0.97–0.99)	t = −3.38, *p* = 0.028
Work pressure	1.06 (0.98–1.15)	t = 2.14, *p* = 0.099
Outcome: Professional Accomplishment		
Moderate		
Drinker	2.87 (1.17–7.36)	t = 3.10, *p* = 0.036
Intended to resign	1.66 (1.10–2.51)	t = 3.41, *p* = 0.027
High		
Drinker	2.01 (0.43–9.45)	t = 1.25, *p* = 0.278
Intended to resign	1.35 (0.53–3.42)	t = 0.89, *p* = 0.424

^a^ Adjusted for the clustering effect.

## Data Availability

Data is not accessible without permission due to the funding requirement. However, de-identified data could be available on a case-by-case basis.
